# Prevalence of pre-eclampsia in women in the Middle East: a scoping review

**DOI:** 10.3389/fpubh.2024.1384964

**Published:** 2024-08-06

**Authors:** Ayatullah Hegazy, Farida Abdelrehim Eid, Farah Ennab, Yrsa Bergmann Sverrisdóttir, William Atiomo, Aida Joseph Azar

**Affiliations:** ^1^College of Medicine, Mohammed Bin Rashid University of Medicine and Health Sciences (MBRU), Dubai Health, Dubai, United Arab Emirates; ^2^Department of Physiology, School of Health Sciences, University of Iceland, Reykjavík, Iceland

**Keywords:** pre-eclampsia, Middle East, scoping, review, pregnancy, UAE, prevalence

## Abstract

Hypertensive disorders of pregnancy are the second most common cause of maternal deaths worldwide. Metabolic syndrome is recognized as one of the risk factors for pre-eclampsia. A recent study revealed a high prevalence of metabolic syndrome in the United Arab Emirates (UAE), particularly amongst Emirati women compared with global estimates. This finding raises the possibility that the prevalence of pre-eclampsia in the region may also be higher as research is increasingly demonstrating an association between pre-eclampsia and metabolic syndrome. We therefore conducted this scoping review of the literature to investigate the nature and extent of studies evaluating the prevalence of pre-eclampsia within the Middle East region to enable subsequent comparison of these findings with the global burden of pre-eclampsia, objectively identify gaps in the literature and inform the design of future studies to address these gaps. PubMed and Scopus were used to extract studies published over the last 20 years (2003–2023). The search terms used included (“Pre-eclampsia” AND “Prevalence”) OR (“Hypertension in pregnancy” AND “Prevalence”) OR (“Pregnancy” AND “Pre-eclampsia”) OR (“Pre-eclampsia” AND “Epidemiology”). We limited our studies to those from the Middle East (ME). A total of 556 relevant articles were identified following which 11 were shortlisted for review. There were four studies from Iran, two from Saudi Arabia, two from Qatar, one from Jordan, and one from Bahrain. The remaining study included 29 countries from Africa, Asia, Latin America, and the Middle East of which data from Jordan, Lebanon, the Occupied Palestinian Territory, and Qatar were included. There were four retrospective, two cross-sectional, and two cohort studies, one prospective study, one meta-analysis, and one descriptive-analytical study. The prevalence of pre-eclampsia in the studies ranged from 0.17 to 5%. We did not find any study investigating the prevalence of pre-eclampsia in the United Arab Emirates. Based on our findings, we conclude that there is a significant scarcity of research in this area, especially within the Middle East, and notably an absence of studies specifically pertaining to the UAE. Consequently, we assert that there is a pressing requirement for additional research to evaluate the prevalence of pre-eclampsia in the region.

## 1 Introduction

Pre-eclampsia, characterized as a hypertensive disorder of pregnancy, has a global prevalence of approximately 1–5% of pregnancies and is recognized as a significant contributor to maternal mortality (1, 2). It is defined by the sudden onset of high blood pressure (with systolic blood pressure > 140 mmHg or diastolic blood pressure > 90 mmHg) in a previously normotensive woman after 20 weeks of gestation, often accompanied by proteinuria, multiorgan dysfunction, and uteroplacental dysfunction (3). Hypertensive disorders of pregnancy (including pre-eclampsia) are the second most common cause of maternal deaths worldwide causing an estimated 62,000–77,000 deaths per year (1).

While hypertension and proteinuria are common indicators in the diagnosis of pre-eclampsia, additional criteria are employed to diagnose women with gestational hypertension who progress to pre-eclampsia (4). Furthermore, the severity of pre-eclampsia can range from mild, even asymptomatic, to its more severe manifestations, such as HELLP syndrome which is characterized by the presence of hemolysis, elevated liver enzymes, and a reduced platelet count. Numerous studies have revealed multiple risk factors associated with an elevated likelihood of developing pre-eclampsia (5). These factors include maternal age, pre-existing hypertension, pre-pregnancy weight, a family history of hypertension or pre-eclampsia, smoking, diabetes mellitus, alcohol consumption, and various others (6–10). Additionally, women affected by pre-eclampsia face an increased risk of developing chronic hypertension, hemorrhagic stroke, hemolysis, renal failure, metabolic syndrome, and various other complications. Moreover, there is a risk of progression to eclampsia, a condition characterized by the abrupt onset of seizures, which significantly escalates the disease's impact and worsens the overall prognosis (11–14). A study conducted on the Qatari population showed that neonatal thrombocytopenia was significantly higher in neonates of women with preeclampsia, which signifies the impact of preeclampsia on neonatal health (15).

In a recent study, Mahmoud et al. revealed a significantly high prevalence of metabolic syndrome in the UAE, particularly amongst Emirati women and Asian non-Arab men, when compared to global estimates within the same age group (16). Moreover, a meta-analysis combining eight studies showed significant association between preeclampsia and developing metabolic syndrome (17). Hence, raising the possibility that the prevalence of pre-eclampsia in the region may also be higher likely owing to the shared risk factors between the two conditions (14, 16).

An initial review of the literature did not identify any prior studies that had estimated the prevalence of pre-eclampsia in the UAE. Local guidelines for diagnosis of pre-eclampsia such as the Emirates Obstetrics and Gynecology Society (EOGS) align with American guidelines (18). However, these may not consider local discrepancies. For this reason, the aim of conducting this scoping review of the literature is to investigate the nature and extent of studies evaluating the prevalence of pre-eclampsia within the Middle East region to enable subsequent comparison of these findings with the global burden of pre-eclampsia, objectively identify gaps in the literature and inform the design of future studies to address these gaps.

## 2 Materials and methods

### 2.1 Study design, search strategy, and selection criteria

A scoping review of the literature was conducted in accordance with the PRISMA-ScR (Preferred Reporting Items for Systematic Reviews and Meta-Analyses Extension for Scoping Reviews) guidelines (19). In order to provide a thorough exploration of the literature, all types of study designs were included (observational, experimental, systematic reviews, and meta-analysis). Our search strategy incorporated specific search terminologies to include all published studies over the last 20 years (January 2003–May 2023) from two primary databases: PubMed and Scopus. Other databases were not included due to limitations in accessibility. The search period was restricted to the past 20 years as it allowed us to capture the most recent and relevant data on the prevalence of preeclampsia in the Middle East. Additionally, narrowing the scope to the last 20 years potentially allowed for a more manageable and focused review, enabling a thorough analysis of the most recent trends, patterns, and factors contributing to the prevalence of preeclampsia in the region. The terms used in our search thread included (“Preeclampsia” AND “Prevalence”) OR (“Hypertension in pregnancy” AND “Prevalence”) OR (“Pregnancy” AND “Preeclampsia”) OR (“Preeclampsia” AND “Epidemiology”). During the literature search process, we were particularly interested in articles assessing the prevalence of pre-eclampsia in women residing in Middle Eastern countries. These countries included Bahrain, Cyprus, Egypt, Iran, Iraq, Israel, Jordan, Kuwait, Lebanon, Oman, Occupied Palestinian Territory, Qatar, Saudi Arabia, Syria, Turkey, United Arab Emirates (UAE), and Yemen. The primary literature search was restricted to articles published in both English and Arabic, as Arabic is the predominant language in the Middle East (ME). Despite including the Arabic language in our on the prevalence of pre-eclampsia in the ME, we found none. Therefore, we focused our research exclusively on articles published in English. Reports and abstracts were excluded. The preliminary data search and strategy was conducted by the Information and Technical services (IT) manager at the investigator's institution on 11th May 2023. To update the search, a repeat search was performed on PubMed on 14th January 2024 by one of the authors (AH) to ensure there were no additional studies following our initial search. The same MeSH terms and countries were used in the repeat search. The search period was January 2003–December 2023. Following a title and abstract screen by two of the co-authors (AH and WA), no additional relevant articles were identified.

### 2.2 Data extraction

**Figure 1:**
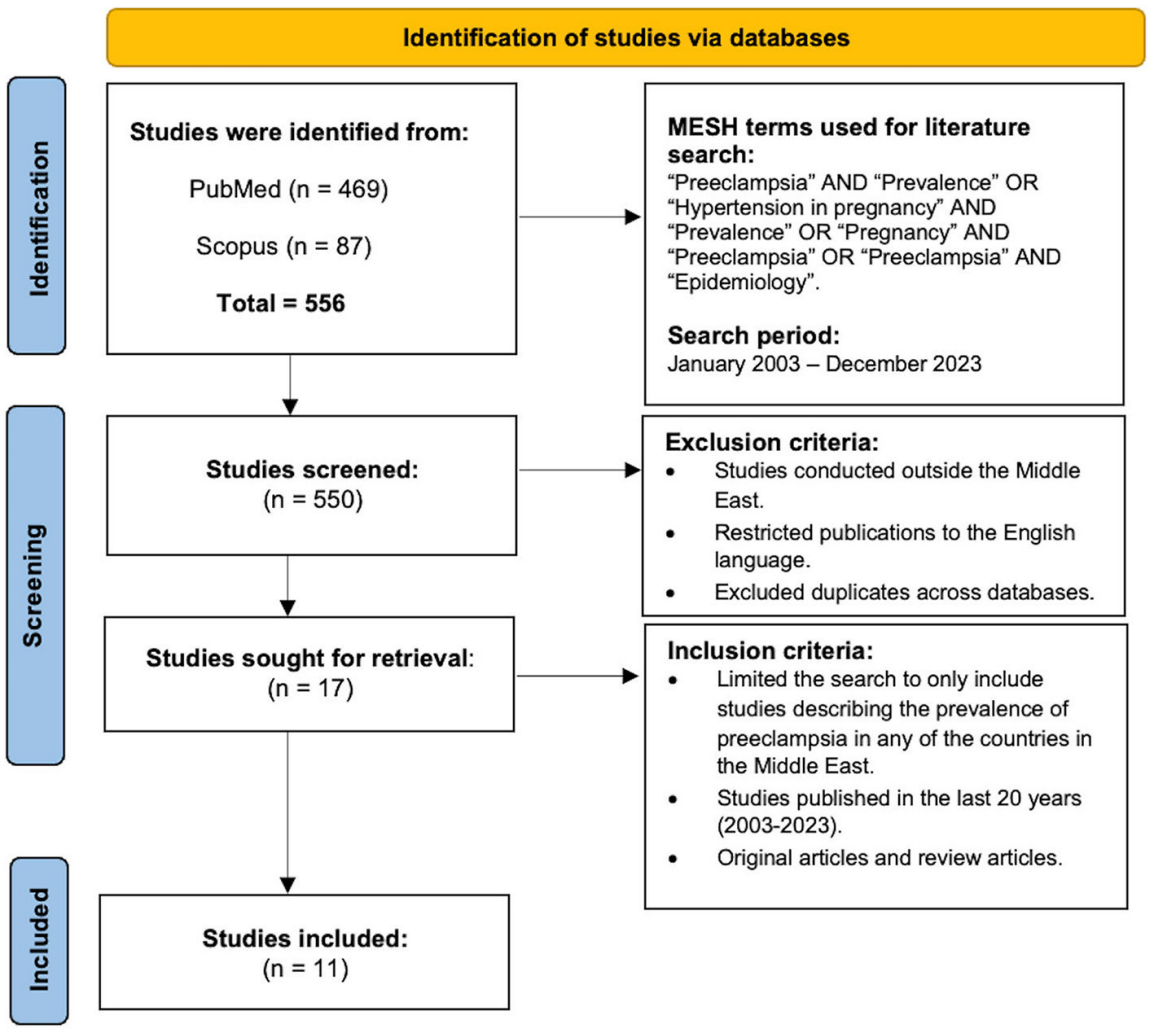
PRISMA chart detailing the results from the literature search and the criteria applied.

Following the initial literature search, two reviewers (AH and FAE) then independently accessed the databases and screened the title and abstracts of all identified articles, exporting pertinent articles into an Excel File Spreadsheet. Upon the completion of data collection, a collective reassessment of all articles was conducted resulting in the exclusion of any article that did not explicitly state the prevalence of pre-eclampsia in the chosen sample, articles published in countries outside the Middle East, articles published in any other language and all duplicates across databases. Upon applying our exclusion criteria (studies outside the Middle East, publications not in the English Language) and removing the duplicates, eligible full-text articles were identified for review and data extraction (Figure 1).

The eligible full-text articles were fully reviewed to extract the following variables: year of publication, country, source of patients, study design, study aims, definition of preeclampsia, data collection time frame, sample size, prevalence, prevalence percentage, non-control and control group. In addition, a column for “other comments” was added to highlight any additional findings that did not fit into the previous criteria. The sum of the reported number of cases was not extracted from the articles, as scoping reviews are intended to provide only a narrative description of the research in question, unlike meta-analyses and systematic reviews, which report the total population size of the accumulated literature. In this scoping review we report the prevalence of preeclampsia as percentages rather than total numbers. This approach facilitates easier interpretation and allows for comparisons in further epidemiological analyses.

### 2.3 OSF registration

This study protocol was registered with the open-source cloud, Open Science Framework (OSF) with details on research design, hypothesis, and method of data collection. https://doi.org/10.17605/OSF.IO/ZGCPE.

### 2.4 Ethical approval

Ethical approval for this student research study was granted by the MBRU, Institutional Review Board (Reference # MBRU IRB-2023-62).

## 3 Results

### 3.1 Description of the studies

Figure 1 shows the PRISMA flowchart of the selection process. A total of 556 relevant articles were identified from PubMed and SCOPUS using the search terms outlined in the methodology section. Following the exclusion criteria of studies outside the Middle East, publications not in the English Language, and duplicates of articles, the abstracts of 550 articles were screened. The inclusion criteria were then applied which included studies describing the prevalence of pre-eclampsia in any Middle Eastern country, published in the last 20 years (2003–2023), including original articles and review articles. The titles and abstract were screened following which 11 eligible full-text articles were found to fulfill the inclusion criteria and were identified for review and data extraction.

The study authors, year of publication, country of study, study design as defined in the article, source of patients and sample size, are listed in Table 1. Iran was the country where most of the studies were conducted (four articles), followed by Saudi Arabia and Qatar with two articles each, while Jordan and Bahrain each featured in one research article. The remaining study (22) included data from 29 countries from Africa, Asia, Latin America, and the Middle East of which data from Jordan, Lebanon, the Occupied Palestinian Territory, and Qatar were included. The study designs, as defined by the article authors, were four retrospective (20, 27–29), two cross-sectional (28, 29), and two cohort (23, 25), one prospective (24), one meta-analysis (30), and one descriptive-analytical study (26).

**Table 1:** Summary of characteristics of included studies.

**References**	**Study year**	**Study location**	**Source of patients**	**Study design^a^**	**Sample size**	**Prevalence of preeclampsia**
Zareian (20)	2004	Iran	Shahid Motahhari Hospital of Jahrom School of Medical Sciences	Descriptive, cross-sectional, retrospective survey	2,300	1.04%
Zibaeenezhad et al. (21)	2010	Iran	Hafez and Zeinnabieh hospitals affiliated to Shiraz University of Medical sciences	Descriptive, prospective, cross-sectional study	24,196	0.17%
Abalos et al.^b^ (22)	2014	29 countries from Africa, Asia, Latin America and the Middle East (*Jordan, Lebanon, OPT and Qatar*)	Health facilities in 29 countries from Africa, Asia, Latin America and the Middle East.	Cross-sectional study (secondary analysis of the World Health Organization Multicountry Survey on Maternal and Newborn Health (WHOMCS) database)	313,030 (*10,138 women from the Middle East)*	Jordan (4.72%), Lebanon (1.02%), OPT (2.35%), Qatar (1.19%).
Wahabi et al. (23)	2016	Saudi Arabia	Khalid University Hospital (KKUH), King Fahad Medical City (KFMC), King Abdul-Aziz Medical City (KAMC)	Cohort study	14,568	1.2%
Khader et al. (24)	2017	Jordan	Ministry of Health, Royal Medical Services, Private sector, and University Hospitals	Prospective study	21,980	1.3%
Sadoun et al. (25)	2017	Qatar	Recruitment of 3,000 mothers through the Ministry of Health in Qatar and conducting baseline questionnaire and face to face interview	Protocol for a birth cohort study	3,000	N/A
Tavakolipoor et al. (26)	2018	Iran	Al-Zahra Hospital Amin Hospital Isa-Ibn-Maryam Hospital Shahid Beheshti Hospital	Descriptive-analytic study	2,477	4.2%
Subki et al. (27)	2018	Saudi Arabia	King Abdulaziz University Hospital	Retrospective study	9,493	1.3%
Maducolil et al. (28)	2021	Qatar	Women's Hospital	Population-based retrospective data analysis	19,762	2.3%
Rajab et al. (29)	2021	Bahrain	Salmaniya Medical Complex and peripheral Maternity Units	Retrospective analytical study	31,639	2.7%
Kharaghani et al. (30)	2023	Iran	N/A^c^	Meta-analysis	132,737	5%

^a^The study design listed in this table represents the study design as defined by the article authors.

^b^Number of women from the Middle East, 10,138 (Jordan 1,166, Lebanon 4,042, Occupied Palestinian Territory 980, Qatar 3,950).

^c^All descriptive, cross-sectional, prospective, or retrospective cohort studies, in which the prevalence or cumulative incidence of pre-eclampsia in the hospitals of Iran was reported, were retrieved regardless of publication date and language.

OPT, the Occupied Palestinian Territory.

The study sample sizes ranged from 2,477 (26) to 132,737 (30), with the largest being a meta-analysis.

### 3.2 Prevalence of pre-eclampsia

The prevalence of pre-eclampsia in the studies included ranged from 0.17% (21) to 5% (30). Maducolil et al. (28), conducted a population-based retrospective data analysis in Qatar where the prevalence rate was 2.30%. In Jordan, Khader et al. (24), carried out a prospective study where the prevalence rate was 1.30%. In Saudi Arabia, Subki et al. (27) conducted a retrospective study where the prevalence rates were 1.30%.

Additionally, Wahabi et al. (23) conducted a cohort study in Riyadh, the capital of Saudi Arabia, where the prevalence was 1.20%. Several studies in Iran contributed to the literature on hypertensive disorders of pregnancy, including Tavakolipoor et al.'s (26) descriptive-analytic study in Isfahan, Zibaeenezhad et al.'s (21) descriptive cross-sectional study in Shiraz, Zareian's (20) retrospective survey in Jahrom and Kharaghani et al.'s (30) meta-analysis which found a prevalence of 4.20, 0.17, 1.04, and 5 % respectively. The World Health Organization's multicounty survey, analyzed by Abalos et al. (22), covered 29 countries across Africa, Asia, Latin America, and the Middle East, exploring pre-eclampsia and eclampsia, and associated outcomes which expressed a prevalence of 2.16%. The countries in the Middle East covered by the survey included Jordan, Lebanon, Occupied Palestinian Territory and Qatar with 10,138 women and prevalence rates of 4.72, 1.02, 2.35, and 1.19%, respectively. A birth cohort study in Qatar, led by Sadoun et al. (25), described a protocol which aimed to evaluate maternal health pre- and postnatally and identify gene-environmental interactions affecting fetal growth however no prevalence rates were mentioned.

## 4 Discussion

There is a lack of prior published research investigating the prevalence of pre-eclampsia in the Middle East. In this scoping review of the literature, 11 articles were identified, assessing the prevalence of pre-eclampsia in women in the Middle East that met the inclusion criteria. The study designs varied from individual hospital-based studies to comprehensive population-based studies and one meta-analysis. However, the studies were undertaken in only five of the seventeen countries encompassing the Middle East. One of the studies covered a broader geographical scope of which four were in the Middle East (Jordan, Lebanon, Occupied Palestinian Territory and Qatar). The prevalence of pre-eclampsia in the studies included ranged from 0.17 to 5%. We did not find any study investigating the prevalence of pre-eclampsia in the United Arab Emirates.

The prevalence of pre-eclampsia identified in this study is consistent with the reported global prevalence which is estimated at between 1 and 5% (1). A significant increase in the prevalence of metabolic syndrome among the young population in the United Arab Emirates was noted compared to the global prevalence estimates (16), highlighting the need for targeted public health interventions. This suggests a possibility that women from the Middle East might have a higher prevalence of pre-eclampsia, as pre-eclampsia is associated with impaired maternal metabolic and cardiovascular function (16). It is however important to note a substantial gap in the results due to the limited amount of research conducted in the Middle East, especially as we did not find any published study specifically from the United Arab Emirates. We also did not find any publicly available data from an established patient bioregistry in the UAE.

We conducted this scoping review after noting that not only is obesity in women of high prevalence in the UAE (31) but also the metabolic syndrome has a significantly high prevalence amongst Emirati women compared to global estimates (16). Mechanistically, it is thought that altered metabolic and cardiovascular function (1) in women contribute to pre-eclampsia by causing reduced spiral artery remodeling in early-onset pre-eclampsia and altered placental metabolic function in both early-onset and late-onset pre-eclampsia (32).

In the UAE, where obesity rates are rising, genetic associations have been identified between single-nucleotide polymorphisms and features of the metabolic syndrome including body mass index, waist circumference and type 2 diabetes mellitus (33). Vitamin D deficiency which is prevalent in the UAE is also associated with metabolic syndrome (34). Variations in the vitamin D receptor gene in Emiratis has also been associated with the susceptibility to type 2 diabetes (35). However, whether this increased mechanistic predisposition to the metabolic syndrome in women in the UAE results in an increased prevalence of pre-eclampsia is unknown.

The primary strength of this study is its novelty, since, to the best of our knowledge, there have been no prior reviews that investigated the prevalence of pre-eclampsia in the Middle East. However, due to the limited number of identified articles, our results may not adequately represent a significant portion of the countries in the region. Additional limitations include our search being restricted to only two databases, the inclusion of studies published in English, and the variability in the design of the included studies. The studies included in our research varied not only in study design but also in sample size and source of patients which can significantly affect the outcomes and conclusions drawn from the data. This highlights challenges faced when generalizing the findings and causes difficulty in assessing the prevalence in the region. Although a comprehensive critical appraisal of the articles is not essential for a scoping review, we acknowledge it has a potential constraint of our study.

In conclusion, our scoping review reveals that there is a scarcity of published research investigating the prevalence of pre-eclampsia in pregnant women within the Middle East, and notably an absence of studies specifically pertaining to the UAE. There is therefore a pressing requirement for additional research to investigate the prevalence of pre-eclampsia in the region, given the higher prevalence of metabolic risk factors, as there is a possibility that it is currently underestimated. Retrospective reviews of electronic health records (36) and analysis of data from ongoing longitudinal birth cohort studies (37) in the UAE provide an opportunity to address this research gap. This is of particular significance, to enhance awareness among both the population and healthcare providers, which would lead to improvements in the screening and diagnosis of pre-eclampsia. This would ultimately reduce the disease burden, minimize complications through early detection and contribute to an overall enhancement in the quality of care received by pregnant women in the region.

## Author contributions

AH: Writing – original draft, Writing – review & editing, Data curation, Formal analysis, Investigation, Methodology, Resources. FAE: Data curation, Investigation, Methodology, Resources, Writing – original draft, Writing – review & editing. FE: Methodology, Resources, Writing – original draft, Writing – review & editing. YS: Conceptualization, Data curation, Investigation, Methodology, Project administration, Resources, Supervision, Visualization, Writing – original draft, Writing – review & editing. WA: Data curation, Formal analysis, Investigation, Methodology, Project administration, Resources, Supervision, Writing – original draft, Writing – review & editing. AA: Funding acquisition, Methodology, Project administration, Resources, Supervision, Writing – original draft, Writing – review & editing.
